# Computational Analysis Reveals a Critical Point Mutation in the *N*-Terminal Region of the Signaling Lymphocytic Activation Molecule Responsible for the Cross-Species Infection with Canine Distemper Virus

**DOI:** 10.3390/molecules26051262

**Published:** 2021-02-26

**Authors:** Yuta Yamamoto, Shogo Nakano, Fumio Seki, Yasuteru Shigeta, Sohei Ito, Hiroaki Tokiwa, Makoto Takeda

**Affiliations:** 1 Department of Chemistry, Rikkyo University, Nishi-Ikebukuro, Toshima, Tokyo 171-8501, Japan; yuta.abinitio.yamamoto@gmail.com; 2 Graduate School of Integrated Pharmaceutical and Nutritional Sciences, University of Shizuoka, 52-1 Yada, Suruga, Shizuoka 422-8526, Japan; snakano@u-shizuoka-ken.ac.jp (S.N.); itosohei@u-shizuoka-ken.ac.jp (S.I.); 3 PREST, Japan Science and Technology Agency, Saitama, Kawaguchi 332-0012, Japan; 4 Department of Virology 3, National Institute of Infectious Diseases (NIID), Musashimurayama 208-0011, Japan; fseki@nih.go.jp; 5 Center for Computational Sciences, University of Tsukuba, 1–1–1 Tennodai, Tsukuba, Ibaraki 305-8577, Japan; shigeta@ccs.tsukuba.ac.jp

**Keywords:** canine distemper virus, signaling lymphocytic activation molecule, fragment molecular orbital calculation, molecular dynamics simulation

## Abstract

Infection of hosts by morbilliviruses is facilitated by the interaction between viral hemagglutinin (H-protein) and the signaling lymphocytic activation molecule (SLAM). Recently, the functional importance of the *n*-terminal region of human SLAM as a measles virus receptor was demonstrated. However, the functional roles of this region in the infection process by other morbilliviruses and host range determination remain unknown, partly because this region is highly flexible, which has hampered accurate structure determination of this region by X-ray crystallography. In this study, we analyzed the interaction between the H-protein from canine distemper virus (CDV-H) and SLAMs by a computational chemistry approach. Molecular dynamics simulations and fragment molecular orbital analysis demonstrated that the unique His28 in the *N*-terminal region of SLAM from Macaca is a key determinant that enables the formation of a stable interaction with CDV-H, providing a basis for CDV infection in Macaca. The computational chemistry approach presented should enable the determination of molecular interactions involving regions of proteins that are difficult to predict from crystal structures because of their high flexibility.

## 1. Introduction

Morbilliviruses belong to the *Paramyxoviridae* family and cause systemic infection of animals with high mortality and morbidity rates [1]. In the infection cycle, the viral hemagglutinin (H) protein interacts with the signaling lymphocytic activation molecule (SLAM) and poliovirus receptor-like 4 (nectin-4), which are expressed on host immune and epithelial cells, respectively [1]. The amino acid sequence of nectin-4 is highly conserved among species, whereas the amino acid sequence of SLAM is not conserved, suggesting that the interaction between the H-protein and SLAM defines host selectivity of morbilliviruses. Currently, seven virus species in the morbillivirus genus have been isolated, including the measles virus (MV) that infects humans [1]. The canine morbillivirus (canine distemper virus, CDV) causes a severe and fatal infection for animals in the *Carnivora* order and has attracted research interest as a target for determining the cross-species transmission of morbilliviruses, particularly because CDV causes lethal outbreaks in non-human primates [2,3,4]. Accumulating evidence indicates that CDV infects animals in the genus *Macaca* but not humans. Interestingly, the macaca SLAM, but not human SLAM, functions as a CDV receptor [4,5]. Moreover, a small number of mutations to the CDV H-protein enable this protein to interact with other primate SLAMs from the *Saguinus* genus (e.g., cotton-top tamarin) and *Homo* genus (human) [6,7,8]. Defining the molecular mechanism responsible for CDV cross-species transmission in primates may be resolved by analyzing the differences among SLAMs from these species, with structural data playing an important role in providing insights into this mechanism.

Many research groups have attempted to determine the crystal structures of the H-protein from morbilliviruses [9,10,11,12], and some structures in complex with receptor proteins have been reported [10,12]. Hashiguchi et al. [10] reported the crystal structure of the H-protein from MV (MV-H) bound to the cotton-top tamarin SLAM, which is known to function as an efficient MV receptor [13,14]. Analysis of the structure and cotton-top tamarin SLAM mutants indicated that residues in the CC’-loop region of the cotton-top tamarin SLAM, such as N72, V74, E75, and K77, form interactions with MV-H [10]. The oligomeric state of MV-H may change upon binding SLAM, suggesting that these changes are associated with triggering fusion of the MV [10]. In contrast, the functional role for the predicted highly flexible *N*-terminal region of SLAM in virus infection remains unknown because this region is missing in the reported crystal structure [10]. The functional importance of this region has been shown by combinational analysis using a virus infection assay and SLAM mutants. Seki et al. [15] reported that the M29S mutant of human SLAM could not interact with MV-H, suggesting that structural analysis of the *N*-terminal region of SLAM is required to fully elucidate the molecular mechanism responsible for the formation of the H-protein-SLAM complex.

As discussed above, the flexible *N*-terminal region may be important for facilitating infection by morbilliviruses. Here, an alternative approach to X-ray crystallography is required to determine the function of the *N*-terminal region of SLAM in CDV infection because structural data of CDV-H and *N*-terminal residues of SLAM are unavailable. To tackle this challenge, we constructed a model of CDV-H complexed with the *N*-terminal region of SLAM. Using the constructed model, interaction energy analysis between the SLAM and CDV-H was performed by a computational chemistry approach. Based on the results, we hypothesize how residues in this *N*-terminal region of SLAM affect the interaction between CDV-H and SLAM at the molecular level.

## 2. Results and Discussion

### 2.1. Protein Sequence Comparison between Human SLAM and Macaca SLAM

**Figure 1:**
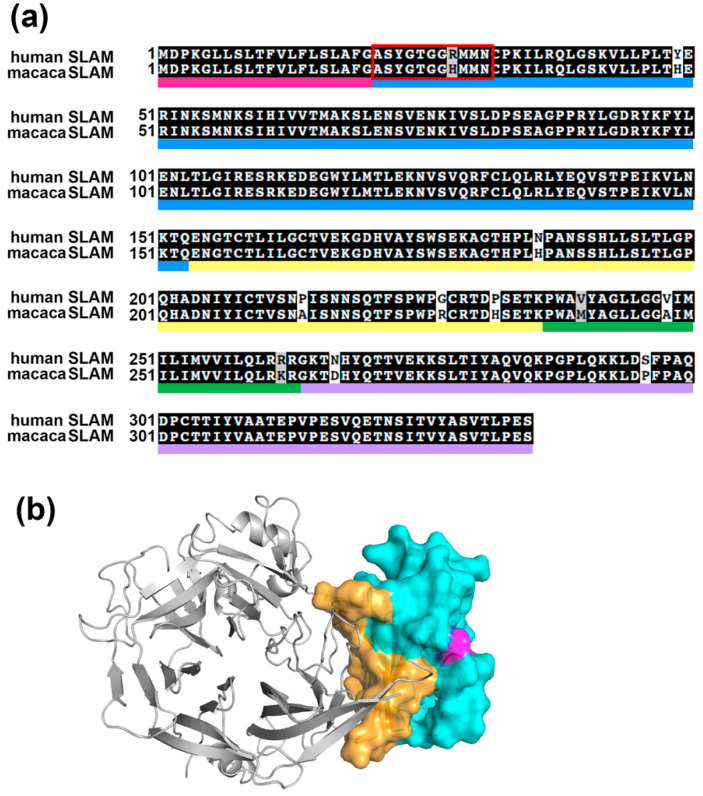
(**a**) Sequence alignment of human signaling lymphocytic activation molecule (SLAM) and macaca SLAM. GenBank accession numbers for human SLAM and macaca (*Macaca mulatta*) SLAM were NP_003028 and XM_001117605, respectively. The alignment was performed by ClustalW. Signal peptides of SLAM were predicted by SignalP. SLAM has five domains: signal peptide (pink), V domain (blue), C2 domain (yellow), transmembrane domain (green) and cytoplasmic domain (purple). (**b**) Overall structure of MV-H (gray cartoon representation) and macaca SLAM (surface representation). Residues at the interaction surface are shown in orange and others are in cyan. Residue 48 (magenta) is not located at the interaction interface (orange).

Initially, we wanted to identify residues that trigger cross-species transmission of CDV among primates because CDV-H has been shown to interact with macaca SLAM but not with human SLAM [4,5,7]. This observation suggests that differences in the amino acid sequences of the two SLAMs are responsible for this difference in affinity toward CDV-H. A sequence alignment of human and macaca SLAMs is shown in Figure 1a, suggesting that only 11 residues are different to each other. In general, SLAM can be divided into five domains: signal peptide (marked in pink in Figure 1a), V domain (blue), C2 domain (yellow), transmembrane domain (green), and cytoplasmic domain (purple). Structural data for MV-H complexed with the cotton-top tamarin SLAM demonstrated that the V domain contributes solely to the interaction with CDV-H. Only residues 28 and 48 in the V domain differ in amino acid type between the macaca and human SLAMs (Figure 1a), suggesting that these two residues may account for the functional difference between human and macaca SLAMs in CDV infection. Residue 48 is located distal from MV-H and SLAM interaction interface (Figure 1b), suggesting that this residue is not important for the interaction. The potential role of residue 28 in forming the complex cannot be determined because residues in the *N*-terminal region of SLAM (red square in Figure 1a) are missing in the structure by Hashiguchi et al. [10]. Moreover, modeling of the *N*-terminal region structure of human SLAM complexed with MV-H suggested that residue 28 plays only a minor role in the interaction with MV-H [15]. Therefore, an approach to predict the structure in the *N*-terminal region of SLAM is necessary for determining the role of residue 28 in the interaction with CDV-H.

### 2.2. Interaction Energy Analysis between CDV-H and SLAM by Fragment Molecular Orbital (FMO) Analysis

The potential functional roles of residues in the *N*-terminal region of SLAM were determined by constructing a homology model of the macaca SLAM in complex with CDV-H by a computational chemistry approach. The model structure of CDV-H complexed with macaca SLAM was initially generated by using Molecular Operating Environment (MOE) software and the crystal structure of MV-H complexed with the cotton-top tamarin SLAM as the template. Sequence identities between MV-H and CDV-H, and the cotton-top tamarin SLAM and macaca SLAM were 35% and 83%, respectively, suggesting that a highly accurate model structure of CDV-H and macaca SLAM can be obtained through modeling. For the modeled CDV-H, we confirmed that the structure was not unfolded after the MD simulation. Then, complementation of residues in the *N*-terminal region was performed by using the structural geometry in this region and scoring calculated by MOE. The structure that had the highest score was selected from the generated models. In addition, we confirmed that the important interactions are conserved in the modeled structures as well as the crystal structure.

**Figure 2:**
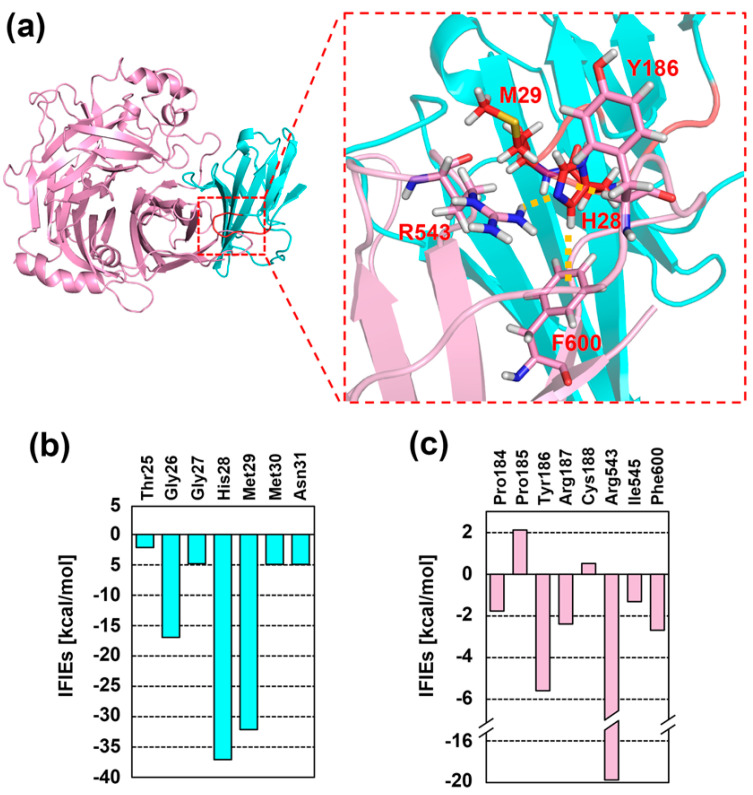
(**a**) Residues at the interaction interface between CDV-H (pink) and macaca SLAM (cyan). (**b,c**) inter-fragment interaction energies (IFIEs) of the interacting residues of macaca SLAM (cyan bar, (**b**) and CDV-H (pink bar, (**c**) by fragment molecular orbit (FMO) analysis.

The interaction interface between the *N*-terminal region of macaca SLAM (red in Figure 2a) and CDV-H (pink in Figure 2a) showed that two residues of macaca SLAM (His28 and Met29) interact with three residues of CDV-H (Tyr186, Arg543, Thr544, and Phe600). This observation was supported by quantitative FMO analysis of the interaction energies. The energies of macaca SLAM are represented as binding affinities in Figure 2b (cyan bar), showing that His28 and Met29 form attractive interactions with CDV-H; the energies for His28 and Met29 were −36.9 and −32.2 kcal/mol, respectively, and these values were >15 kcal/mol lower than energies for other residues (Figure 2b). Calculation of the inter-fragment interaction energies (IFIEs) between His28 and residues in CDV-H revealed that Tyr186, Arg543, and Phe600 of CDV-H form interactions with macaca SLAM (Figure 2c). The IFIEs for Tyr186, Arg543, and Phe600 were −5.6, −19.8 and −2.7 kcal/mol, respectively.

In summary, His28 contributes significantly to the formation of the CDV-H-macaca SLAM complex. In particular, interactions between His28 and three CDV-H residues (Tyr186, Arg543 and Phe600) are the main contributors that enable the formation of this complex.

### 2.3. Molecular Dynamics Simulation of the CDV-H and Macaca SLAM Complex

The static structure analysis described above indicated that His28 and Met29 of macaca SLAM play key roles in the formation of a stable complex with CDV-H. To validate this observation, the dynamics of the CDV-H-macaca SLAM complex was examined by molecular dynamics (MD) simulations. MD simulations of CDV-H complexed with the wild-type (WT) macaca SLAM (WT), macaca SLAM H28R variant, and M29S variant were performed. Human SLAM has an arginine at position 28.

**Figure 3:**
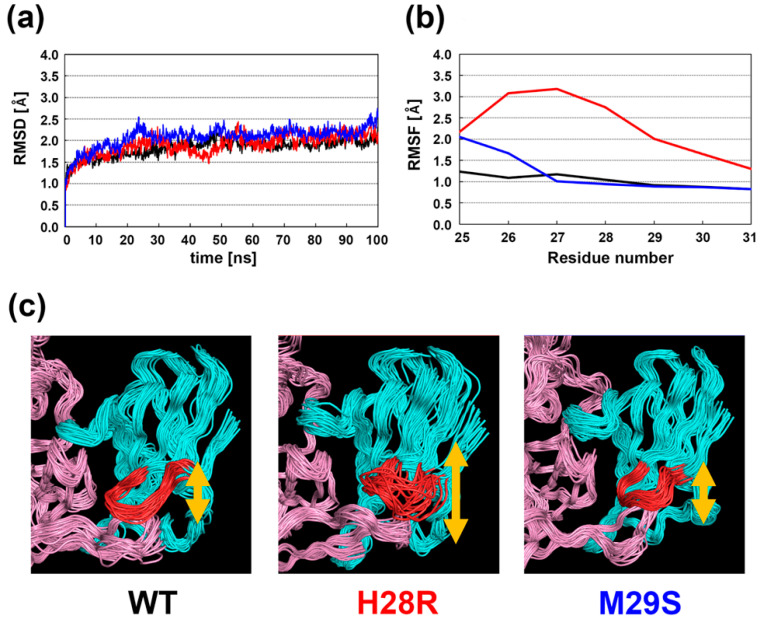
Molecular dynamics (MD) simulations of the CDV-H-macaca SLAM complex. (**a**) RMSD values for Cα atoms during the 100 ns MD simulations. The RMSD values for WT and the H28R and M29S variants are shown in black, red, and blue, respectively. (**b**) RMSFs for residues (from 25 to 31) in the *N*-terminal region of macaca SLAM. The color code is the same as in (**a**). (**c**) Trajectory structures of the CDV-H‒macaca SLAM complex. The *N*-terminal region is more flexible than other regions of the complex.

Initially, root-mean-square deviation (RMSD) values for Cα atoms were determined, and these values indicated that the structures had equilibrated after 30 ns simulation (Figure 3a) with RMSD values of approximately 2.2 Å. This result indicated that the MD simulations can be performed correctly. Next, structural changes at the *N*-terminus of macaca SLAM (residues 25 to 31) during the simulation were analyzed by calculating root mean square fluctuation (RMSF) values for Cα atoms (Figure 3b). The analysis showed that only the H28R mutation of macaca SLAM increases the flexibility of the region; the RMSF values for residues 27‒29 of the H28R variant were >2.0 Å (Figure 3b, red), whereas the values for WT (black) and M29S (blue) were <1.3 Å (Figure 3b). Analysis of the trajectory structures also supported this observation. As shown in Figure 3c, flexibility in the *N*-terminal region of macaca SLAM H28R was clearly higher than those of the WT and M29S variant (Figure 3c). MMGBSA analysis showed that the H28R mutation decreased the interaction energy between macaca SLAM and CDV-H by ~20 kcal/mol when compared with those of WT and M29S (Table 1).

In summary, quantitative analyses of static and dynamic structures of CDV-H and macaca SLAM showed that the interaction formed between His28 of macaca SLAM and residues of CDV-H is essential for the formation of a stable macaca SLAM-CDV-H complex. In detail, the high stability in macaca SLAM-CDV-H would be brought by forming interaction between side chain H28 and each of Y186, R543 and Y600 (Figure 2c). The interactions would be disappeared by H28R mutations, and this brings to highly flexibility at the *N*-terminal region. Here, computational analysis of H28R variant was performed to show the difference between hSLAM and macSLAM (Figure 1a); a similar phenomenon may be observed in H28K mutant.

## 3. Materials and Methods

### 3.1. Homology Modeling

Structures of the complexes were constructed using the MOE program [16]. The crystal structure of the MV-H-SLAM complex (PDB ID: 3ALW) was used as the initial template [10]. This structure is composed of the MV-H head (amino acids 184‒607) and the SLAM-V (amino acids 30‒140) domains, and the two domains are linked by a 12-residue flexible linker (Gly-Gly-Gly-Ser)_3_. Thus, the structure has the following two missing elements: (1) *N*-terminus of SLAM (including the important residue 28) and (2) part of the *N*-terminus of MV-H. The incomplete *N*-terminal regions of MV-H and SLAM were modeled by using the Loop Modeler utility of MOE to obtain the complete MV-H-SLAM complex structure. For this, sequences of the MV IC-B strain (GenBank accession number NC_001498) for the MV-H protein part [a partial *N*-terminal region available with the published MV-H structure (PDB ID: 2ZB6 [9]) was also utilized] and the macaca SLAM for the SLAM part (GenBank accession number XM_001117605) were used along with the initial MV-H‒SLAM template (PDB ID: 3ALW [10]). This complete model, ^comp^MV-macaca SLAM, was used as the template for structural modeling of the CDV-H-macSLAM complex. The sequence of CDV Ac96I strain (GenBank accession number AB753775) for the CDV protein part, along with the newly constructed ^comp^MV-macSLAM structure, as a template, was used to model the structure of the CDV-H-macSLAM complex. Missing hydrogen atoms were added using the Protonate 3D utility of MOE using the AMBER10:EHT force-field with the solvation energy determined by the Born model. The resulting structures were fully optimized with the AMBER10:EHT force-field. The structures of the H28R and M29S SLAM variants in complex with CDV-H were modeled by using the constructed WT CDV-H‒SLAM structure and the Protein Builder utility of MOE. All structures were visualized by PyMOL Molecular Graphics System, Version 2.0 Schrödinger, LLC.

### 3.2. Molecular Dynamics Simulations

The initial setups for the MD simulations were carried out using AMBER14 [17] and ff14SB force-field [18]. The constructed structures of the complexes were solvated with the TIP3P water model in a 110 × 90 × 90 Å^3^ cubic box. Neutralizing counter ions were added to each system. AMBER topology files created using AMBER were converted to GROMACS format using the acpype.py script [19]. All MD simulations were performed using the GROMACS package [20]. Bonds with H atoms in the constructed structures were treated as rigid bodies by using the LINCS algorithm [21]. To equilibrate the system, an 800 ps NPT simulation was performed using the Nose-Hoover thermostat [22,23] at 300 K by keeping heavy atoms constrained and this was followed by a 100 ns NPT simulation with the Parrinello–Rahman method at 1 bar and 300 K [24,25]. For non-local interactions, the electrostatic interactions were calculated with the particle mesh Ewald method using a real-space cutoff of 10 Å. The cutoff value for van der Waals interactions was set to 10 Å.

### 3.3. Binding Free Energy Calculations

The molecular mechanics generalized Born surface area (MM-GB/SA) approach [26] implemented in AMBER14 was used to calculate the binding free energy for all simulated systems involved in our MD calculations. A total of 100 conformations were extracted from the last 20 ns of the MD simulations. The MM-GBSA calculations were carried out after removing water molecules and counterions. The enthalpy term (H) was computed using the modified GB model developed by A. Onufriev et al. [27]. The concentration of 1–1 mobile counterions in solution was set to 0.15 M. The entropy term (‒TΔS) was computed under the Nmode module in AMBER14 utilizing default values.

### 3.4. RMSD and RMSF Calculations

Root-Mean-Square Deviation (RMSD) and Root-Mean-Square Fluctuation (RMSF) calculations were carried out by using the cpptraj [28] analysis tool of AMBER14. Structures were sampled at 10 ps intervals. Before performing each calculation, external translational and rotational motions were removed by minimizing the RMSD distance of the Cα atoms to the equivalent atoms in the first frame of the trajectory. RMSD and RMSF values were calculated for Cα atoms.

### 3.5. Fragment Molecular Orbital (FMO) Calculations

FMO calculations were carried out using the PAICS program [29]. The correlated Resolution-of-Identity second-order Moller Plesset (RI-MP2) level of theory [29] with correlation consistent double-zeta basis set cc-pVDZ was used for the calculations. The fragment assignment and PAICS input generation were performed using PaicsView [30]. Outputs were analyzed using RbAnalysisFMO [31].

Concisely, the FMO method, a first-principles calculation-based quantum mechanical method, is a powerful theoretical tool to reliably study protein-ligand interactions. In FMO calculations, each structure of a protein-ligand complex is divided into one-residue fragments with cutoff points at the Cα atom of each residue, and the ligand is also considered as a fragment and the properties of the whole system are derived in a many-body expansion by combining the properties of the fragments. By considering two-body systems (fragment pairs), the inter-fragment interaction energies (IFIEs) can be calculated, which are important physical quantities for understanding protein-ligand binding. In the present study, FMO was used to study protein-protein interactions. FMO is currently used as a valuable tool for describing protein-ligand interactions [32].

## 4. Conclusions

**Figure 4:**
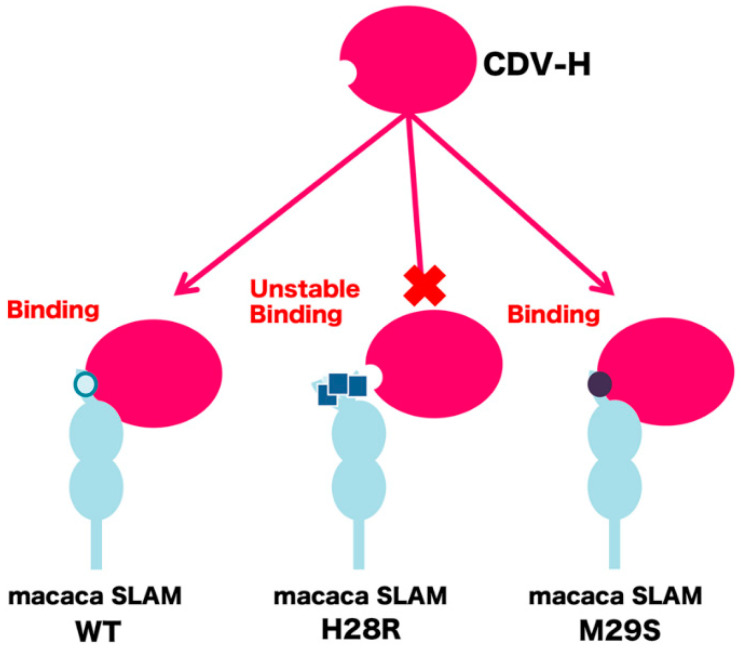
Schematic showing how key residues in the *N*-terminal region of macaca SLAM contribute to the interaction with CDV-H.

The molecular basis for differentiated receptor functions between macaca SLAM and human SLAM for CDV infection has not been investigated in detail. In this report, we proposed a detailed and specific model of the molecular mechanism with which the *N*-terminal region of macaca SLAM forms the stable interaction with CDV-H. The results obtained in this study are summarized in Figure 4, showing that only the H28R mutation destabilizes the interaction. We have shown previously [15] the validity of the simulation approach in analyzing the interaction between human SLAM and MV-H. This previous study combining computational and experimental approaches showed that Met29, which is located in the *N*-terminal region of human SLAM, is essential for forming the interaction with MV-H. Taken together, our computational approach should facilitate analysis of molecular interactions that cannot be determined by crystal structure analysis because the protein regions that form the interaction interface are highly flexible.

## Figures and Tables

**Table 1:** Interaction energies between macaca SLAM and CDV-H by MMGBSA analysis.

	∆G [kcal/mol]
WT	−31.8
H28R	−7.2
M29S	−30.9

## Data Availability

Not applicable.

## Sample Availability

Samples of the compounds are not available from the authors.
